# Fever Burden Is an Independent Predictor for Prognosis of Traumatic Brain Injury

**DOI:** 10.1371/journal.pone.0090956

**Published:** 2014-03-13

**Authors:** Long Bao, Du Chen, Li Ding, Weihua Ling, Feng Xu

**Affiliations:** Department of Emergency medicine, the First Affiliated Hospital of Soochow University, Suzhou, China; The Ohio State University Medical Center, United States of America

## Abstract

**Objective:**

To evaluate fever burden as an independent predictor for prognosis of traumatic brain injury (TBI).

**Methods:**

This retrospective study involved 355 TBI patients with Glasgow Coma Scale (GCS) ≤14, who presented at the emergency department of our hospital between November 2010 and October 2012. At 6 months follow-up, patients were divided into 5 groups based on Glasgow Outcome Scale (GOS) and dichotomized to GOS score (high (4 to 5) vs. low (1 to 3)). The relationship between fever burden and GOS was assessed.

**Results:**

Fever burden increased as GOS scores decreased from 5 to 2, except for score 1 of GOS, which corresponded to a significant lower fever burden. Following dichotomization, patients in the high GOS group were younger, and showed less abnormal pupil reactivity (*P*<0.001), a higher median GCS score (*P*<0.001), and a lower median fever burden (*P*<0.001), compared with patients in the low GOS group. Univariate logistic regression analysis revealed that poor TBI prognosis was related to age, GCS, pupil reactivity, and fever burden (OR: 1.166 [95% CI: 1.117–1.217] *P*<0.0001). Multivariate logistic regression analysis identified fever burden as an independent predictor of poor prognosis after TBI (OR 1.098; 95% CI: 1.031–1.169; *P* = 0.003). These observations were confirmed by evaluation of the receiver operating characteristic (ROC) curve for fever burden (area under the curve [AUC] 0.73 [95% CI: 0.663–0.760]).

**Conclusion:**

Fever burden might be an independent predictor for prognosis of TBI. High fever burden in the early stage of the disease course associated with TBI could increase the risk of poor prognosis.

## Introduction

Traumatic brain injury (TBI) is a common cause of injury, death, and disability in people younger than 40 years. Over 100 TBI prognostic models have been reported. However, over 90% of TBI occurs in developing countries, but only 2% of the TBI models are based on patients from these countries [1]. In China, the incidence of TBI has recently increased due to the increasing popularity of high-speed motor vehicles and adventure sports, and the rapid development of the construction industry. In fact, it is predicted that TBI will be the third most common cause of death in 20 years [2].

The prognosis of TBI varies depending on the type and location of the injury, the associated pathology, and the severity of lesions (quantified using the Glasgow Coma Scale (GCS)). Therefore, precise and valid outcome predictions are difficult [3]. To date, three approaches to outcome prediction following TBI are available: the first is based on admission characteristics, including age, Glasgow Coma Scale (GCS) score, pupil reactivity, blood glucose levels, and presence of major extracraninal injury; the second is represented by the Marshall computed tomographic (CT) classification, and is based on pathological findings seen on the first available CT scan; and the third uses serum or cerebrospinal fluid (CSF) biomarker levels [4].

Recently, a prognosis calculator was developed using the International Mission for Prognosis and Clinical Trial (IMPACT) and the Corticosteroid Randomization After Significant Head Injury (CRASH) databases, which contain information from large randomized controlled trials and epidemiological studies. The ten strongest positive predictive factors were identified and included in three prognostic models, but only the ‘core model’ was associated with a considerable predictive value. Constituents of the ‘core model’ are age, motor score component of the GCS, and reaction of the pupils [5].

Fever is a common condition in patients with brain injuries and may occur in 20–50% of TBI patients [6]. Evidences suggest that more than 50% of TBI patients in the neurological intensive care unit (ICU) experience body temperatures above 38.5°C, and that 68% of TBI patients in the ICU experience at least one episode of fever. This fever in TBI patients may result from multiple causes, and not only from infection. More importantly, fever may be due to disruption of the hypothalamic set point by endogenous pyrogen released from damaged neurons [7].

Previous studies indicated that fever following TBI was associated with poor outcomes in patients with neurological injury [7]. Early fever (within 24 h) has been associated with an increased relative risk of a poor outcome by 2.2-fold with every 1°C increase [8], and even a 0.5°C increase may lead to a series of secondary injuries and neuron death. Poor outcomes may be due to excitotoxicity, free radical production, cytoskeletal proteolysis, inhibition of protein kinases, blood-brain barrier breakdown, or electrolyte disturbance, all of which are exacerbated by fever. In clinical practice, fever is associated with a longer ICU stay, increasing the burden of TBI to both individuals and society.

Previous studies have quantified fever burden (time of >37°C) to evaluate the role of fever prophylaxis as a mean to attenuate secondary injury in TBI patients [9], and to associate cumulative fever burden (daily highest core temperature minus 100.4°F, summed from admission through day 13) with outcomes in patients with subarachnoid hemorrhage [10].

In this present study, we defined fever burden as a parameter representing the severity and duration of fever. We investigated the significance of fever burden for outcomes prediction in TBI. We hypothesized that fever burden is a strong positive predictive factor for prognosis in TBI patients, which may warrant its inclusion as an added parameter in a ‘core prognostic model’ that includes age, GCS score, and pupil reactivity. Furthermore, we evaluated the prognostic value of fever burden for TBI through a comparison with other predictors.

## Materials and Methods

### Patients

This was a retrospective study of 355 consecutive patients (253 men and 102 women; mean age of 49.1±24.9; range: 19–66 years) who visited the emergency department or intensive care unit (ICU) of our hospital between November 2010 and October 2012 with TBI and a GCS score ≤14. Inclusion criteria were: 1) traumatic brain injury within 24 hours; 2) GCS score 3–14; and 3) no sedative drugs, muscle relaxant or tracheal intubation before admission to the ICU or emergency department. Patients <18 years old, pregnant women, and patients with an autoimmune disease were excluded. Patients' characteristics, including age, pupil reactivity, and GCS score after stabilization of respiration and hemodynamics were collected. The study protocol was approved by the Ethics Committees of the First Affiliated Hospital of Soochow University (Suzhou, China) and all participants and/or a relative provided a written informed consent.

### Fever burden

Patients were hospitalized at the ICU or at the normal ward. Therefore, body temperature measurements were not performed according to the same schedule (e.g. every two hours for GOS 1 patients, and every six hours for GOS 5 patients). Therefore, because of the retrospective nature of the study, patients' highest body temperature (axillary temperature) recorded on each day for 2 weeks following the injury was used to calculate fever burden. Fever burden was defined as a body temperature >37°C, and was quantified as the highest axillary temperature reached during the day minus 37°C. The total fever burden was defined as the arithmetic sum of the fever burdens during the 14 days, expressed as °C-days [11]. For patients who died within the first 14 days, the total fever burden was the sum of the fever burden while being alive.

### Fever treatments

Fever was treated by physical cooling (bath with warm water or armpit ice bag) for patients whose body temperature was less than 38.5°C. Otherwise, indomethacin or dexamethasone was administrated if the temperature was higher than 38.5°C.

### Prognosis evaluation

Our centre is specialized in TBI and it is standard procedure to follow up patients for at least 6 months after TBI using telephone interviews or medical examinations (outpatient clinics or hospitalized patients). Each follow-up examination or interview was recorded in the medical chart. According to these data, patients were divided into 5 groups based on the GOS scores: 1: death; 2: vegetative state, unable to interact with the environment; 3: severe disability, unable to live independently; 4: moderate disability, able of independent living but unable to return to work or school; and 5: complete recovery, able to work or to attend school. However, the self-perception of full recovery by patients with craniocerebral trauma may not be exactly the same as ‘recovery to their state before trauma’: patients are usually satisfied with the recovery when it allows them to continue their job or studies, and are therefore considered as GOS score 5. The correlation between fever burden and GOS scores was analyzed. Furthermore, patients were dichotomized into two groups: high GOS scores (4 to 5) and low GOS scores (1 to 3). The relationship between fever burden and GOS was assessed.

### Statistical analysis

Categorical data are presented as frequency and proportion (%), and continuous data with normal distribution or non-normal distribution are presented as mean ± SD or median *M (Q_25_, Q_75_)*, respectively. The possible TBI prognostic factors identified from previous studies (age, pupil reactivity, GCS and fever burden) were first analyzed using univariate analyses. Factors that were significant on univariate analyses were entered into a multivariate logistic regression model. Odds ratios (OR) and 95% confidence intervals (CI) were calculated. The correlation between fever burden and GOS score was evaluated using the Spearman correlation analysis. Pairwise comparison of fever burden across GOS scores was performed using the Kruskal-Wallis test. A receiver operating characteristic (ROC) curve was plotted for each patient, and the area under the curve (AUC) was used to assess the prognostic value of each parameter. Stata 12 (StataCorp, Texas, USA) and MedCalc 11 (MedCalc Software, Mariakerke, Belgium) were used for statistical analysis and ROC curves, respectively. *P*<0.05 was considered as statistically significant (two-tailed).

## Results

**Figure 1:**
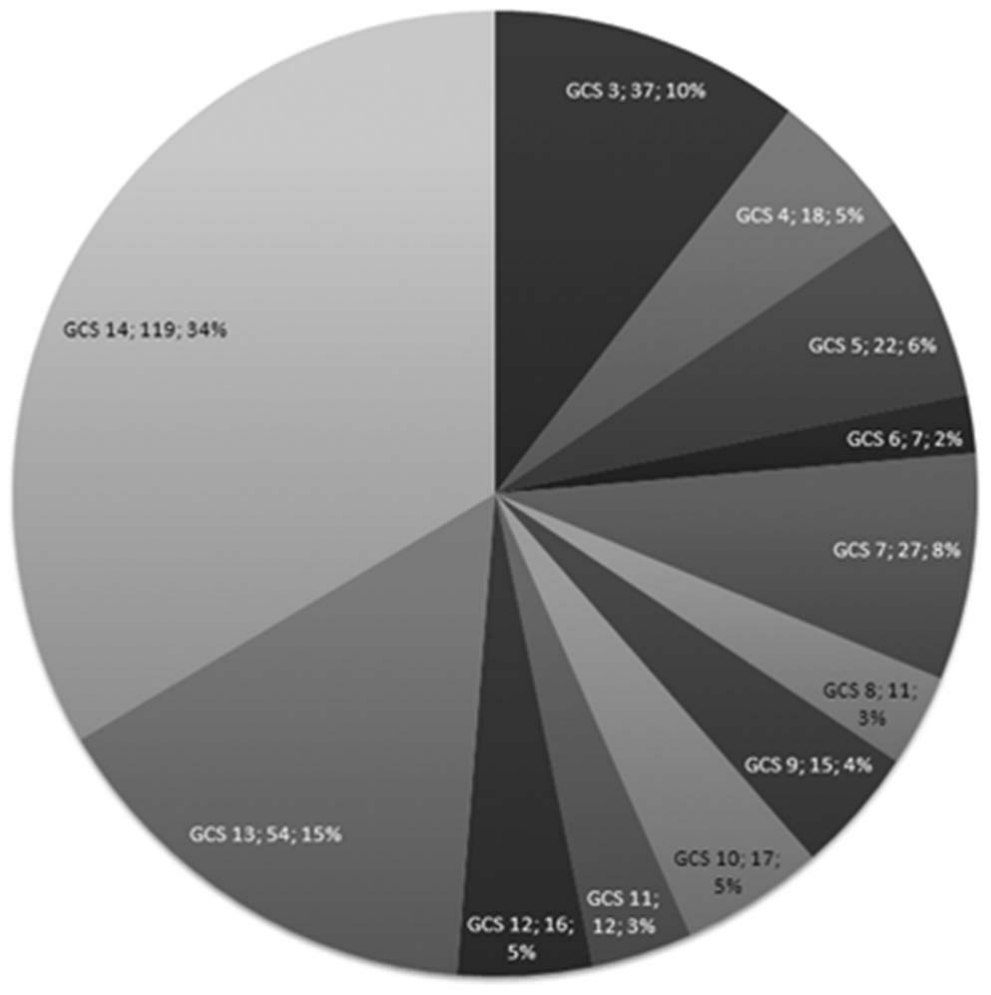
GCS scores distribution of the 315 TBI patients.

**Figure 2:**
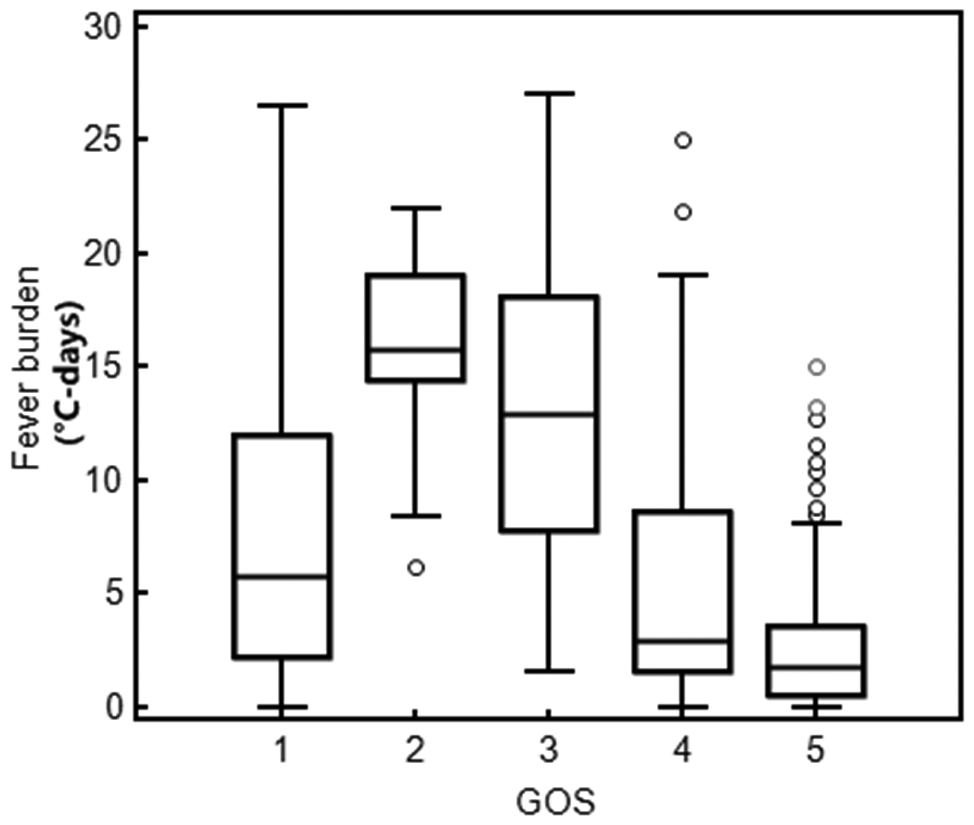
Association between fever burden and GOS (1–5 groups). Note: GOS = 1 n = 101 median: 5.8, Q_25_, Q_75_: 2.2 to 12; GOS = 2, n = 10, median: 15,75, Q_25_, Q_75_: 14.4 to 19.1; GOS = 3, n = 18, median: 12.95, Q_25_, Q_75_: 7.8 to 18.1; GOS = 4, n = 84, median: 2.9, Q_25_, Q_75_: 1.6 to 8.6; GOS = 5, n = 142, median:1.8, Q_25_, Q_75_: 0.5 to 3.6.

Figure 1 shows the distribution of GCS scores in the study patients. Correlation analysis indicated that fever burden increased as GOS scores decreased from 5 to 2 (Figure 2). At GOS 1, the fever burden was significantly decreased, possibly due to the death of most patients during the early stages of their injury, which reduced cumulative fever burden to less than 14 days. Fever burden in GOS 2 patients was significantly different from GOS 1 and GOS 5 patients (P = 0.02 and P<0.001, respectively). Fever burden in GOS 3 patients was significantly different from GOS 4 and GOS 5 patients (P = 0.004 and P<0.001, respectively). Fever burden in GOS 4 patients was significantly different from GOS 2, GOS 3 and GOS 5 patients (P = 0.001, P = 0.004 and P = 0.001, respectively). Finally, fever burden in GOS 5 patients was significantly different from GOS 1, GOS 2, GOS 3 and GOS 4 patients (P<0.001, P<0.001, P<0.001 and P = 0.001, respectively).

Following dichotomization, patients grouped according to high and low GOS scores were significantly different with respect to gender, mean age, pupil reactivity, GCS score, and fever burden (*P*<0.05). Patients in the high GOS group were younger (*P*<0.001) and showed a higher proportion of males, less abnormal pupil reactivity (*P*<0.001), a higher median GCS score (*P*<0.001), and a lower median fever burden (*P*<0.001), compared with patients in the low GOS group (Table 1).

**Table 1:** Characteristics of patients dichotomized by GOS at 6 months follow-up.

	Group favorable prognosis	Group unfavorable prognosis	Statistical magnitude	*P*
	(GOS:4–5, n = 226)	(GOS:1–3, n = 129)		
Gender (n, %)			8.47	0.004
Men	173 (76.55)	80 (62.02)		
Women	53 (24.35)	49 (37.98)		
Age (mean±SD, yrs)	45.0±16.4	56.2±34.1	−4.16	<0.001
Pupil reactivity at admission (n, %)			154.44	<0.001
Bilateral response	212 (93.81)	44 (34.11)		
Unilateral response	13 (5.75)	29 (22.48)		
Bilateral no response	1 (0.44)	56 (43.41)		
GCS score at admission (median, Q_25_, Q_75_)	13 (12, 14)	5 (3, 8)	12.76	<0.001
Fever burden* (median, Q_25_, Q_75_)	2 (0.7, 4.9)	7.8 (3.2, 14.5)	−6.73	<0.001

*: Sum of body temperature over 37°C from day 1 to 14 during the hospitalized.

Univariate logistic regression analysis revealed that poor TBI prognosis was closely related to age, GCS, pupil reactivity, and fever burden (*P*<0.001). The OR associated with fever burden was 1.166 (95% CI: 1.117–1.217) (Table 2).

**Table 2:** Association between prognostic predictors and outcome analyzed by logistic regression.

Predictor	Univariate logistic regression		Multivariate logistic regression	
	OR value (95% CI)	*P*	OR value (95% CI)	*P*
GCS score	0.609 (0.556–0.667)	<0.001	0.718 (0.618–0.833)	<0.001
Pupil reactivity	12.707 (7.200–22.426)	<0.001	5.063 (2.196–11.676)	<0.001
Fever burden	1.166 (1.117–1.217)	<0.001	1.098 (1.031–1.169)	0.003
Age	1.028 (1.015–1.041)	<0.001	1.065 (1.041–1.089)	<0.001

Multivariate logistic regression analysis identified fever burden as an independent predictor of TBI (OR 1.098; 95% CI: 1.031–1.169; *P* = 0.003) after adjustment, and suggests that for an increase of one unit of fever burden, the risk of an unfavorable prognosis was raised by 9.8%.

**Figure 3:**
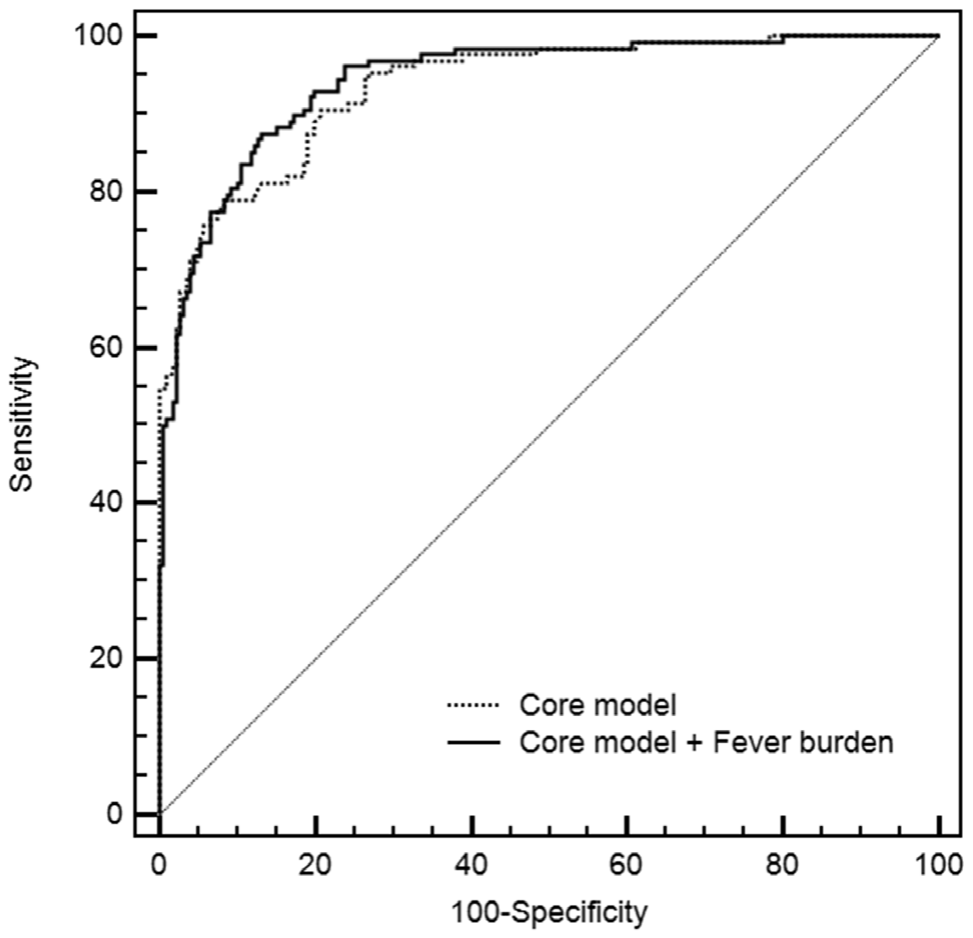
ROC curves of the core model and of the core model+fever burden.

Characteristics of the ROC curve for fever burden included an AUC of 0.73 (95% CI: 0.663–0.760), which was lower than the AUC for GCS and pupil reactivity, but higher than the AUC for age (Table 3). These data confirm the prognostic value of fever burden and place its positive predictive value between age and GCS and pupil reactivity. When fever burden was added into the core prognostic model, the R^2^ was increased from 0.5345 to 0.5506, suggesting that adding our estimation of fever burden to the core model slightly improved the prognosis of the model (Figure 3).

**Table 3:** Receiver operating characteristic curve analysis for prognostic predictors.

	AUC	SE	95% CI
GCS	0.897	0.0187	0.861 to 0.927
Pupil reactivity	0.809	0.0225	0.764 to 0.849
Fever burden	0.713	0.0312	0.663 to 0.760
Age	0.642	0.032	0.590 to 0.692

Fever burden was negatively correlated with GOS score (r = −0.376, 95%CI: −0.462 to −0.283, *P*<0.001), further suggesting that high fever burden is indicative of a risk of an unfavorable prognosis following TBI.

## Discussion

Our findings strongly suggest that age, GCS and pupil reactivity are precise and valid predictors for prognosis in TBI patients. Our study identified fever burden as an independent prognostic predictor for TBI, and suggested that fever burden might be a critical parameter for prediction of outcomes in TBI patients.

Prognostic models of TBI are of value for both clinical practice and academic research. Accurate assessment of prognosis based on clinical findings and robust evidence is essential to therapeutic decision-making, for counseling patients and relatives, for comparing the efficacy of TBI treatment across multiple healthcare settings, and for the evaluation of treatment outcomes associated with therapies in clinical trials [4].

Currently, there are two authoritative online prognostic systems, both of which are based on characteristics at admission only and do not take into account the evolution of the patient in time. The system derived from the IMPACT database [12] consists of three models: the core model (predictors: age, GCS score, and pupil reactivity), the extended model (core model predictors combined with hypoxia, hypotension, CT Marshall score, tSAH, and epidural hematoma), and the laboratory model (extended model predictors combined with glucose and hemoglobin levels). The system developed from the CRASH database [13] includes multiple predictors such as nationality, age, GCS score, pupil reactivity, major extracranial injury, and pathological findings from CT scans. The IMPACT and CRASH models share several parameters including age, GCS score (or GCS motor score), and pupil reactivity.

The factors included in the core model are already well-known prognostic factors for TBI. Older age is a factor associated with poorer outcomes following brain injury [14]–[17]. The GCS was initially used as an objective assessment of coma and impaired consciousness [18]. Subsequently, it has been widely used to evaluate the severity of TBI and its relation to prognosis [15]. However, accurate assessment of the GCS may be compromised by the use of sedative drugs and muscle relaxants [19]. In the present study, included TBI patients did not receive medical treatment such as sedative drugs, muscle relaxants, or tracheal intubation until admission, which allowed valid assessment of the prognostic value of the GCS. Finally, abnormal pupil reactivity usually indicates compression or injury in the brain stem, and is significantly associated with unfavorable prognosis [15].

The development of the IMPACT models focused on moderate and severe TBI patients, while the CRASH models also included patients with milder injuries [5]. In the present study, patients with GCS scores between 3 and 14 were selected, and a ‘core model’ of prognostic prediction, including age, GCS score, and pupil reactivity was adopted for assessment of TBI prognosis. Our findings were consistent with previously published results. However, our data indicate that prognostic prediction is more precise and valid when fever burden is included as a prediction parameter.

Fever is common in critically ill patients with neurological and neurosurgical diseases, and is closely associated with unfavorable prognosis. A recent report revealed that fever is an independent risk factor that may increase ICU and hospital stay, as well as mortality in these patients, when confounding factors such as severity of disease, diagnosis, age and complications are controlled for [9]. Results from pairwise analyses showed that the fever burden in patients with milder injury (GOS 5) was significantly less than in any of the other group, except GOS 1 patients. However, because many of these patients died during the early course of their disease, fever burden data was underestimated. Therefore, our results suggest that fever burden is associated with the severity of TBI.

The Copenhagen Stroke Study group showed that a 1°C decrease in body temperature could almost double the rate of a favorable prognosis (OR, 1.8). Reith *et al* found that a 1°C increase in body temperature could elevate the risk of poor prognosis by 2.2 times [20]. Therefore, these studies underline the importance of body temperature in the prognosis of TBI.

The concept of fever burden was proposed during the 1980' and 1990', and was calculated as the product of the fever and its duration. It was used as one parameter for the evaluation of a catheter-based heat exchange system in critically ill neurologic and neurosurgical patients [21]. Fever burden quantitatively represents the effect of fever severity and duration. Indeed, a long-lasting, more severe fever may result in a greater fever burden, increased adverse events, and poorer outcomes. In the present study, the fever burden of TBI patients within two weeks after injury was retrospectively analyzed to evaluate its association with prognosis. Our findings showed that fever burden was negatively associated with GOS, and that fever burden was demonstrated to be an independent prognostic factor for TBI by univariate logistic regression, multivariate logistic regression, ROC curve analysis, and correlation analysis.

However, the use of fever burden as a prognostic factor for TBI is limited. First, because the present study was retrospective, all patients did not undergo the necessary procedures to allow the determination of Marshall scores. Second, it was found that the fever burden of patients with a GOS of 1 was lower than that of patients with a GOS of 2. This may be because patients with severe TBI (GCS = 3) had a shorter survival after injury, resulting in the accumulation of fever burden over fewer than 14 days, or these patients may be more likely to experience hypothermia due to severe stress. Third, we measured axillary temperature, which may differ from brain temperature. Indeed, a precise brain temperature would be more valuable for prognosis assessment of TBI. Fourth, the use of a single high temperature recorded in a 24 hour period may not adequately describe burden of fever. Indeed, a patient who is febrile for 12 of 24 hours has a significantly higher fever burden than a patient who is febrile for 2 of 24 hours, but the design of our study could not address this. Finally, the main limitation was the retrospective nature of our study: some of our patients (for example, GOS 1) in ICU got their body temperature measured every two hours, while some patients (for example, GOS 5) in the ordinary ward got their body temperature measured every six hours. Therefore, since body temperature measurements were not comparable between patients, we chose to use the highest temperature of the day minus 37° as the day fever burden. A more precise fever burden assessment could be made based on the hourly fever burden, which could maybe increase the R^2^ of the model. This will be the focus of a future prospective study.

Regardless of the causes of fever or the methods to treat fever, the present study focused on the consequences of fever burden, and the correlation between fever burden and prognosis, rather than the relationship between the therapeutic treatment against fever and prognosis. Indeed, even if treatments are provided for the fever, the fever burden remains if body temperature does not decrease enough, and will still affect prognosis.

In conclusion, the addition of fever burden to the parameters used in the current core prognosis prediction model (age, pupil reactivity and GCS score) may be expected to provide a more precise and valid measure of outcomes in TBI patients. However, further prospective multicenter studies are required to confirm these results and to assess the cause-effect relationship between fever control and outcomes of medical management of TBI patients.
